# Osilodrostat: A Novel Potent Inhibitor of 11-Beta-Hydroxylase for the Treatment of Cushing's Syndrome

**DOI:** 10.17925/EE.2024.20.1.8

**Published:** 2023-12-11

**Authors:** Rosario Pivonello, Chiara Simeoli, Nicola Di Paola, Angelica Larocca, Erminio Massimo Crescenzo, Annamaria Colao

**Affiliations:** 1. Dipartimento di Medicina Clinicae Chirurgia, Sezione di Endocrinologia, Diabetologia, Andrologiae Nutrizione, Università “Federico II” di Napoli, Naples, Italy; 2. UNESCO Chair for Health Education and Sustainable Development, University Federico II, Naples, Italy

**Keywords:** Cushing's disease, Cushing's syndrome, medical treatment, osilodrostat, steroidogenesis inhibitor

## Abstract

Osilodrostat is a novel potent oral steroidogenesis inhibitor with a non-steroidal chemical structure, recently approved for the treatment of adult patients with endogenous Cushing's syndrome, and Cushing's disease not cured bytab pituitary surgery or in whom pituitary surgery is not an option. Osilodrostat has been evaluated in different multicentre phase II and III clinical studies, and has shown to have notable effects, such as significant reductions in cortisol secretion, associated with significant improvement in body weight, blood pressure, glucose metabolism, lipid profile, psychological status and quality of life. The favourable safety profile, combined with the relevant efficacy, could make osilodrostat suitable as medical treatment in several phases of the Cushing's syndrome treatment journey: before surgery, as preoperative treatment, or instead of surgery, in cases where surgery is not an option or refused, as first-line treatment; after surgery, in cases of persistent or recurrent disease, as second-line treatment; after second surgery or radiotherapy following pituitary surgery as bridging treatment waiting for the definitive disease control, as third-line treatment. Further real-world clinical experience data are needed to confirm the current knowledge.

Osilodrostat, a novel potent oral steroidogenesis inhibitor, has recently been approved for the treatment of adult patients with endogenous Cushing's syndrome (CS), and Cushing's disease (CD) not cured by pituitary surgery or in whom pituitary surgery is not an option. Osilodrostat acts by inhibiting the adrenal enzymes 11-beta-hydroxylase and aldosterone synthase, and by inducing the decrease of cortisol and aldosterone production. Osilodrostat displays a longer half-l ife and a higher potency compared with those of the two classical adrenal steroidogenesis inhibitors, metyrapone and ketoconazole, and may be administered twice daily, at relatively lower dosages, to reach the same efficacy.

Phase II and III trials have demonstrated that osilodrostat induces a potent, rapid and effective disease control, associated with cardiovascular, metabolic, and quality of life improvements; it maintains sustained efficacy in the long-term follow-up, without reported escape phenomenon.^1–6^ Moreover, phase II and III trials have demonstrated that osilodrostat has a good safety profile, characterized by the occurrence of common adverse events (AEs), mainly including decreased appetite, nausea, diarrhoea, fatigue, headache and arthralgia, with AEs of special interest, such as hypocortisolism-related AEs, adrenal hormone precursor accumulation-related AEs, QT interval prolongation and pituitary tumour enlargement.

This mini review summarizes the available data illustrating the promising role of osilodrostat for the treatment of CS, provides general notes on pharmacodynamics and pharmacokinetics, and overviews the different multicentre clinical studies that evaluated drug efficacy and safety, supporting the employment of the agent in the treatment of CS. It includes a final discussion aimed at highlighting key practical clinical considerations and recommendations for the use of this drug.

Osilodrostat has recently achieved an emerging role in the medical treatment of CS based on the relevant efficacy and safety data, which demonstrated a rapid control of cortisol secretion in a consistent number of cases, with a sustained improvement in the clinical picture and a good safety profile.^7,8^

In 2020, osilodrostat was approved by the European Medicines Agency for the treatment of adult patients with endogenous CS, and by the United States Food and Drug Administration for the treatment of adult patients with CD not cured by pituitary surgery or in whom pituitary surgery is not an option.^7,8^

**Figure 1:**
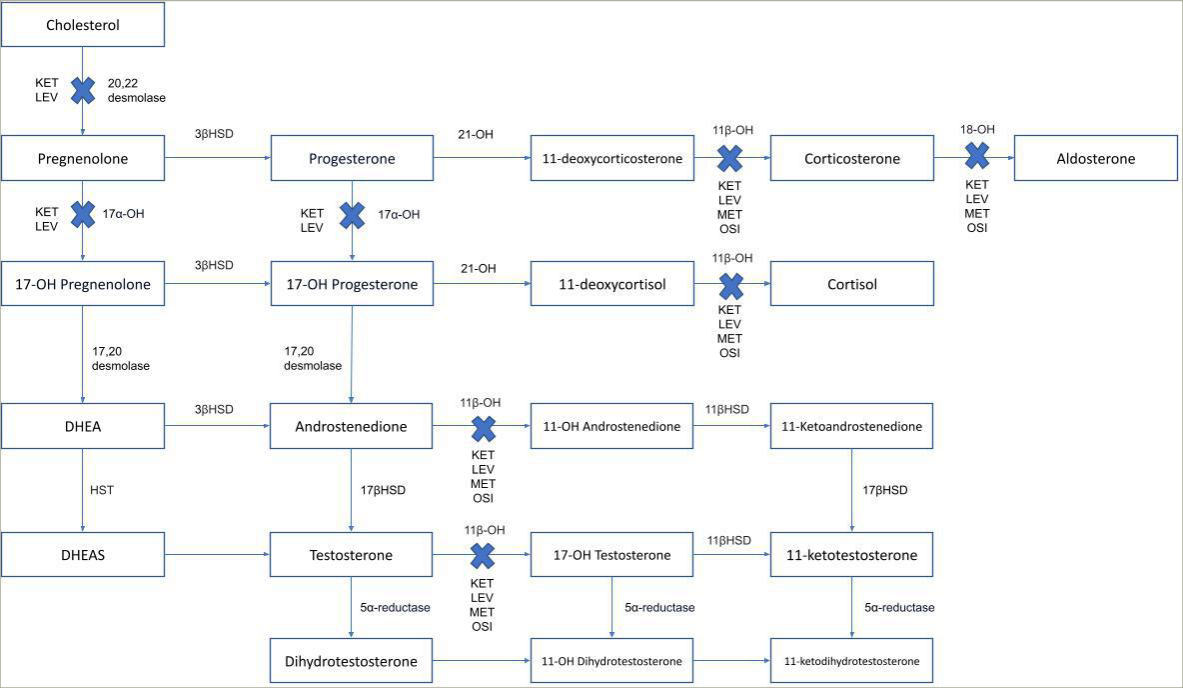
The steroidogenic pathway and mechanism of action of osilodrostat, ketoconazole, levoketoconazole and metyrapone

*Dotted line: minor contribution; bold line: major contribution; blue crosses: enzyme's blockade.*

*DHEA = dehydroepiandrosterone; DHEAS = dehydroepiandrosterone sulfate; HST = hydroxysteroid sulfotransferase; KET = ketoconazole; LEV = levoketoconazole; MET = metyrapone; OSI = osilodrostat; 3βHSD = 3β-hydroxysteroid dehydrogenase; 11βHSD = 11β-hydroxysteroid dehydrogenase; 11β-OH = 11β-hydroxysteroid dehydrogenase; 11-OH androstenedione = 11-hydroxyandrostenedione; 11-OH dihydrotestosterone = 11-hydroxydihydrotestosterone; 11-OH testosterone = 11-hydroxytestosterone; 17α-OH = 17α-hydr oxylase; 17βHSD = 17β-hydroxysteroid dehydrogenase; 17-OH pregnenolone = 17-hydroxypregnenolone; 17-OH progesterone = 17-hydroxyprogesterone; 18-OH = 18-hydroxylase; 21-OH = 21-hydroxylase.*

Osilodrostat, similar to other steroidogenesis inhibitors, can be considered in several instances: as a valid preoperative treatment, especially in cases of severe disease and/or when a rapid hypercortisolism control is required; instead of surgery, as a first-l ine treatment option in cases where surgery is not an option or refused; after surgery, as a second-line treatment option, in cases of persistent or recurrent disease; after a second surgery or radiotherapy following pituitary surgery as bridging treatment waiting for the definitive disease control; or as a third-l ine treatment option.^7^

## Pharmacodynamics and pharmacokinetics

*Figure 1* shows the steroidogenic pathway and mechanism of action of osilodrostat. Known and discovered as an anti-h ypertensive drug, osilodrostat potently acts by inhibiting the adrenal enzyme 11-beta-hydroxylase which catalyses the conversion of 11-deoxycortisol into cortisol, androstenedione into 11-OH androstenedione, testosterone into 11-OH testosterone, and 11-deoxycorticosterone into corticosterone. Moreover, it acts by inhibiting the adrenal enzyme aldosterone synthase, which catalyses the conversion of corticosterone into aldosterone. These effects induce the decrease in cortisol and aldosterone production. Concomitantly, the increase of 11-deoxycortisol, 11-deoxycorticosterone and corticosterone potentially induces or worsens hypertension, peripheral oedema and hypokalaemia.^7–9^ Moreover, the increase of androstenedione and testosterone potentially induces or worsens acne and hirsutism.^9,10^ However, compared with the classical adrenal steroidogenesis inhibitor, metyrapone, which shares a similar mechanism of action to osilodrostat, the increase in 11-deoxycortisol, androstenedione, and testosterone in females has been found to be lower with osilodrostat, suggesting that osilodrostat may be associated with a lower prevalence of hyperandrogenism compared with metyrapone.^11^ This hypothesis may be due to a stronger 17α-hydroxylase, and potentially cholesterol side chain cleavage enzyme and/or steroidogenic acute regulatory protein inhibition of osilodrostat compared with metyrapone.^12^

Osilodrostat displays a higher potency compared with the two classical adrenal steroidogenesis inhibitors, metyrapone and ketoconazole, based on an experimental direct comparison.^9,12^ In an experimental setting assessing half maximal inhibitory concentration (IC50) measurements, osilodrostat inhibits cortisol production more potently than both metyrapone (IC50 0.0347 µM compared with 0.0678 µM) and ketoconazole (IC50 0.0347 µM compared with 0.6210 µM) in human adrenocortical HAC15 cell cultures, suggesting that relatively lower doses of osilodrostat, compared with metyrapone and ketoconazole, may be sufficient to reach the same efficacy.^9,12^

Osilodrostat is absorbed with a time to maximum concentration of ~1 hour and shows a longer half-l ife (~4 hours) compared with those of metyrapone (~2 hours) and ketoconazole (~3 hour 18 minutes), allowing a twice daily administration schedule.^7–9,13–17^

Specifically, based on the European Medicine Agency and United States Food and Drug Administration summaries of product characteristics and prescribing information, osilodrostat is recommended to be initiated at 2 mg orally twice daily (4 mg/day), up to a maximum of 30 mg twice daily (60 mg/day).^17,18^ Some patients, especially those with a mild hypercortisolism, may benefit from a lower starting dose of 1 mg twice daily (2 mg/day), or only at night.^7,16^ A reduced starting dose of 1 mg twice daily (2 mg/day) is also recommended in Asian patients, since their relative bioavailability is ~20.0% higher compared with that of non-Asian patients. Body weight is not a major determinant of this difference.^18^

If a dose is missed, osilodrostat should be administered regularly at the next scheduled time, without changing the dose.^17,18^ Osilodrostat can be administered with or without food, as the delay of time to maximum concentration from 1 hour to 2 hours 30 minutes, observed after a high-fat meal, has not been considered clinically significant.^17^

Osilodrostat should be used with caution when co-administered with cytochrome (CYP) 3A4 inhibitors, and CYP3A4 and/or CYP2B6 inducers, frequently used for the treatment of concomitant CS infectious and neuropsychiatric complications.^17^ Indeed, considering that concomitant use of osilodrostat with a strong CYP3A4 inhibitor, such as itraconazole and clarithromycin, may cause an increase in osilodrostat concentration, a half dose of osilodrostat is suggested.^17^ Conversely, considering that concomitant use of osilodrostat with strong CYP3A4 and/or CYP2B6 inducers, such as carbamazepine, rifampin and phenobarbital, may cause a decrease in osilodrostat concentration and thus reducing its efficacy, a strict monitoring of cortisol concentration and an increase in osilodrostat dosages may be needed.^17^

Multiple CYP enzymes (such as CYP3A4, CYP2B6 and CYP2D6) and uridine diphosphate-glucuronosyltransferases contribute to osilodrostat metabolism.^17,18^ However, the metabolites do not contribute to the pharmacological effect of osilodrostat.

Initially, the dose can be gradually titrated by increments of 1 or 2 mg twice daily (2–4 mg/day) every 1–2 weeks, based on the individual response in terms of 24 hours urinary free cortisol (UFC) changes, individual tolerability, and improvement in signs and symptoms of CS.^17,18^ Subsequently, once the maintenance dosage is achieved, less frequent monitoring may be considered, evaluating cortisol levels at least every 2 months or as indicated by individual clinical response, unless additional monitoring is recommended.^17,18^ The registration of UFC levels below the lower limit of normal (LLN), the occurrence of an excessively rapid decrease in cortisol levels, and/or the appearance of a clinical syndrome suggestive of hypocortisolism require down-titration or temporary discontinuation of osilodrostat.^17,18^ If necessary, glucocorticoid replacement therapy should be initiated. If treatment is interrupted at a certain dose, restarting osilodrostat at a lower dose is recommended when cortisol levels are within target ranges and the clinical syndrome of hypocortisolism has been resolved.^17,18^

No data are available on the use of osilodrostat in pregnancy to assess drug-associated risks of major birth defects, miscarriage or adverse maternal or fetal outcomes. Similarly, there is a lack of data on its use in breastfeeding to evaluate for a drug-associated risk for breastfed infants or milk production.^17^ Notably, for females of a reproductive age, a pregnancy test is recommended before starting osilodrostat treatment. Moreover, for females of child-bearing potential, the use of highly effective methods of contraception during osilodrostat treatment is recommended, and for at least one week after completion.^18^ No data are currently available on osilodrostat use in paediatric patients.^17^ Nonetheless, a paediatric phase II study (Pharmacokinetic (PK), Pharmacodynamic (PD) and Tolerability of Osilodrostat in Pediatric Patients With Cushing's Disease; ClinicalTrials.gov identifier: NCT03708900) is ongoing and is expected to be completed in 2023.^19,20^

Based on the available data for the use of osilodrostat in patients over the age of 65, no dosage adjustment is required.^17^ Furthermore, no dosage adjustment is required in cases of renal impairment.^17^ No dosage adjustment is required in cases of mild hepatic impairment (Child-Pugh A), whereas, in cases of moderate hepatic impairment (Child-P ugh B), the recommended starting dose is 1 mg twice daily (2 mg/day), and in cases of severe hepatic impairment (Child-Pugh C), the recommended starting dose is 1 mg once daily (1 mg/day) in the evening, with a gradual increase up to 1 mg twice daily (2 mg/day).^17,18^

## Clinical evidence

Osilodrostat treatment has been investigated in patients with CD in the two phase II, proof-of-concept, open-l abel, non-randomized, single group assignment studies, LINC 1 and LINC 2, and in the two phase III, prospective, multicentre studies, LINC 3 and LINC 4.^1–4^ Focusing on phase III studies, the LINC 3 study was a double-blind, randomized withdrawal period following a single-arm, open-l abel, dose titration and treatment period; all patients initially received open-l abel osilodrostat, thereafter patients eligible to be randomized, were randomly assigned (1:1) to either continue osilodrostat or receive the matching placebo.^3^ The LINC 4 study was an open-l abel period following a double-blind, randomized, placebo-controlled period; all patients were initially randomly assigned (2:1) to either osilodrostat or placebo, then subsequently all patients received open-l abel osilodrostat.^4^ Moreover, osilodrostat treatment has been investigated in Japanese patients with CS in another phase II, open-l abel, prospective, multicentre study, as well as in patients with CS due to adrenocortical carcinoma and in a retrospective series in comparison with metyrapone.^21–23^

*Table 1* shows the characteristics of studies of osilodrostat for the treatment of CD and CS in terms of study type, patient number, dosages, mean UFC normalization, clinical improvements, AEs and AEs of special interest.^1–6,21–23^

### Studies in patients with Cushing's disease

#### The LINC 1 phase II study

The first phase II, proof-of-concept, prospective, open-l abel, multicentre study, LINC 1 (Safety and Efficacy of LCI699 in Cushing's Disease Patients; ClinicalTrials.gov identifier: NCT01331239) enrolled 12 patients with CD who had undergone pituitary surgery but not pituitary irradiation.^1^ The primary endpoint was the proportion of patients with mean UFC≤upper limit of normal (ULN), defined as complete response, or who had a mean UFC>ULN, but with a ≥50.0% decrease from baseline, defined as partial response, at Day 70 (Week 10). At Week 10, osilodrostat (at dosages of 4–100 mg/day) induced a complete response in 11 (91.7%) and a partial response in one (8.3%) of 12 patients, with an overall control in 100.0% of patients. The treatment with osilodrostat was associated with improvement in blood pressure, although the decrease in systolic and diastolic values was not clinically relevant. Focusing on the androgen production, mean serum testosterone levels significantly increased in the eight females, reaching levels >ULN after 8 weeks of treatment, with no reported AEs of hirsutism and acne, whereas, in the four males, mean serum testosterone levels increased, although non-significantly, from baseline values below the normal range to the LLN, with a new appearance in one (25.0%) case of acne.^1^

Regarding the safety profile, fatigue (58.3%), nausea (41.7%), diarrhoea (25.0%), headache (25.0%), hypokalaemia (25.0%), muscle spasms (25.0%) and vomiting (25.0%) were the most frequently reported AEs. Noteworthy, 33.3% of patients reported AEs consistent with adrenal insufficiency (AI) and/or glucocorticoid withdrawal syndrome. No treatment discontinuation was reported.^1^

**Table 1:** The characteristics of studies of osilodrostat for the treatment of Cushing's disease and Cushing's syndrome^1–6,21–23^

Study	NCT identifier	Study type	Patient number (CS or CD)	Dosages (mg/day)	mUFC normalization	Clinical improvements	AES	AEs of special interest
Bertagna et al.^1^	NCT01331239	OL; P; MC; phase II	12 (CD)	4-100	91.7%	Blood pressure	Fatigue (58.3%), nausea (41.7%), diarrhoea (25.0%), headache (25.0%), hypokalaemia (25.0%), muscle spasms (25.0%), vomiting (25.0%)	NA
Fleseriu et al.^2^	NCT01331239	OL; P; MC; phase II	19 (CD)	4-60	84.2% (W 10); 78.9% (W 22)	Blood pressure, glucose metabolism, lipid profile	Nausea (31.6%), diarrhoea (31.6%), asthenia (31.6%), Al (31.6%)	Adrenal hormone precursor accumulation-related AEs (63.2%), mainly represented by acne (10.5%), hirsutism (10.5%), hypertension (10.5%), hypertrichosis (10.5%) and hypokalaemia (10.5%), together with hypocortisolism-related AEs (57.9%), QT prolongation (5.3%), arrhythmogenic-potentially-related AEs (5.3%)
Fleseriu et al.^5^	NCT01331239	OL; P; MC; phase II	16 (CD)	4-60	75.0% (W 70)	Blood pressure, glucose metabolism	Al (43.8%), arthralgia (37.6%), headache (37.6%), diarrhoea (31.3%)	
Pivonello et al.^3^	NCT02180217	RW, DB following OL; P; MC; phase III	137 (CD)	4-60	86.1% versus 29.4% (W 34); 52.6% (W 24); 66.4% (W 48)	Body weight, blood pressure, glucose metabolism, lipid profile, quality of life, psychological status	Nausea (41.6%), headache (33.6%), fatigue (28.5%), Al (27.7%)	Hypocortisolism-related AEs (51.1%), adrenal hormone precursor accumulation-related AEs (42.3%), mainly represented by hypokalaemia (13.1%) and hypertension (12.4%), together with QT prolongation (3.6%), pituitary tumour enlargement (2.9%), arrhythmogenic-potentially-related AEs (0.7%)
Fleseriu et al.^6^	NCT02180217	RW, DB following OL; P; MC; phase III	106 (CD)	4-60	81.1% (W 72)	Body weight, blood pressure, glucose metabolism, lipid profile, quality of life, psychological status	*Nausea (45.3%), headache (36.5%), fatigue (32.8%), Al (29.2%)	Adrenal hormone precursor accumulation-related AEs (58.4%), mainly represented by hypertension (17.5%), peripheral oedema (16.1%), hypokalaemia (13.1%) and increased blood testosterone (11.7%), together with hypocortisolism-related AEs (54.0%), pituitary tumour enlargement (16.1%)
Gadelha et al.^4^	NCT02697734	OL following R, DB; P; MC; phase III	73 (CD)	4-60	77.1% versus 8.0% (W 12); 80.8% (W 36); 68.5% (W 48)	Body weight, blood pressure, glucose metabolism, lipid profile, quality of life, psychological status	Decreased appetite (45.2%), arthralgia (45.2%), fatigue (38.4%), nausea (37.0%)	Adrenal hormone precursor accumulation-related AEs (61.6%), mainly represented by increased blood testosterone (24.7%) and hypertension (21.9%), together with hypocortisolism-related AEs (27.4%), QT prolongation and/or arrhythmogenic potential (4.1%)
Tanaka et al.^21^	NCT02468193	OL; MC phase II	9 (CS)	4-60	66.7% (W 12); 22.2% (W 24); 11.1% (W 48)	Waist circumference, blood pressure, glucose metabolism, lipid profile	Al (77.8%), gamma-glutamyl transferase increase (33.3%), malaise (33.3%), nasopharyngitis (33.3%)	Hypocortisolism-related AEs (77.8%), adrenal hormone precursor accumulation-related AEs (33.3%), mainly represented by hypokalaemia (22.2%), weight increase (11.1%)
Tabarin et al.^22^	NA	Real-life	7 (CS)	2-60	28.6% (within 1 W); 14.3% (within 1 M); 28.6% (~2 M); 28.6% (3 M)	Blood pressure, glucose metabolism	Al (42.9%), worsening of hypokalaemia (14.3%)	NA
Detomas et al.^23^	NA	Real-life	5 (CD) 3 (CS)	3-20	42.9% (W 4); 50.0% (W 12)	Blood pressure	Depression (12.5%), asthenia (12.5%), nausea (12.5%)	Hypokalaemia (50.0%) and QT prolongation (12.5%)

*Considering the entire study, inciuding the core and the extension periods*

*AE = adverse event; Ai = adrenai insufficiency; CD = Cushing's disease; CS = Cushing's syndrome; DB = doubie-biinded; M = month; MC = muiticentre; mUFC = mean urinary free cortisol; NA = not available; NCT = National Clinical Trial; OL = open-label; P = prospective; R = randomized; RW = randomized-withdrawal; W = week.*

#### The LINC 2 phase II study

The second phase II, proof-of-concept, prospective, open-l abel, multicentre study, LINC 2 (ClinicalTrials.gov identifier: NCT01331239), representing the LINC 1 study expansion by protocol amendment, enrolled 19 patients with CD; four patients completed LINC 1 (the follow-up cohort) and 15, of whom 13 had undergone pituitary surgery and one had undergone pituitary irradiation, were newly enrolled (the expansion cohort).^2^ The primary endpoint was the complete response or the partial response at Weeks 10 and 22. At Week 10, osilodrostat (at dosages of 4–60 mg/day) induced a complete response in 16 (84.2%) and a partial response in one (5.3%) of 19 patients, with an overall control in 89.5% of patients. Whereas, at Week 22, osilodrostat induced a complete response in 15 (78.9%) of 19 patients, with no partial response. The treatment with osilodrostat was associated with improvement in blood pressure, glucose metabolism and lipid profile. Focusing on the androgen production, mean serum testosterone levels significantly increased in the 14 females, presenting testosterone levels >ULN during the study; in the 12 females reaching Week 22, nine (75.0%) had testosterone levels >ULN at the end of the study. New or worsening hirsutism in two cases and/ or acne in three cases were reported among four (28.6%) females during the study, all of whom had testosterone levels >ULN at the end of the study. In males, mean serum testosterone levels increased from baseline values below the normal range to the normal range. Regarding the safety profile, nausea (31.6%), diarrhoea (31.6%), asthenia (31.6%) and AI (31.6%) were the most frequently reported AEs. Treatment discontinuation was reported in two (10.5%) patients during the first 10 weeks as a result of: non-treatment-related administrative issue in one (5.25%) case and AEs in the other (5.25%) case; no treatment discontinuation was reported between Weeks 10 and 22.^2^

The optional extension phase of the LINC 2 study (ClinicalTrials.gov identifier: NCT01331239) enrolled 16 patients with CD, who at Week 22 had mean UFC≤ULN, or were considered by study investigators to be receiving clinical benefit from treatment.^5^ The primary endpoint was the complete or partial response at Week 70. At Week 70, osilodrostat (at dosages of 4–60 mg/day) induced a complete response in 12 (75.0%) and a partial response in one (6.3%) of 16 patients, with an overall control in 81.3% of patients. Notably, long-term data demonstrated that, up to Month 70, the mean UFC response rate for the 16 patients remained between 50.0% and 88.0%. Improvement in blood pressure and glucose metabolism was observed during the entire study, obtained during the core period, and maintained during the extension period. Focusing on the androgen production in females, mean serum testosterone levels, after an initial increase noted at Week 22, decreased back toward baseline levels within the normal range at the last assessment. No new or worsening cases of hirsutism were reported during the extension period. In males, mean serum testosterone levels remained within the normal range during the extension period. Regarding the safety profile, AI (43.8%), arthralgia (37.6%), headache (37.6%) and diarrhoea (31.3%) were the most frequently reported AEs.^5^

Considering the entire study, including the core and the extension periods, AEs were also grouped into categories of special interest, including: adrenal hormone precursor accumulation-related AEs (63.2%), mainly represented by hypertension (10.5%), hypokalaemia (10.5%), acne (10.5%), hirsutism (10.5%) and hypertrichosis (10.5%); hypocortisolism-related AEs (57.9%); QT prolongation (5.3%); and arrhythmogenic-potentially-related AEs (5.3%). Treatment discontinuation was reported in eight (50.0%) patients as a result of: consent withdrawal in two (12.5%) cases, AEs in two (12.5%) cases and a personal decision not to continue treatment in one (6.25%) case. Osilodrostat was no longer required in three (18.75%) cases.^5^

#### The LINC 3 phase III study

The first phase III, double-blind, randomized withdrawal, placebo-controlled following an open-l abel period, prospective, multicentre study, LINC 3 (Safety and efficacy of LCI699 for the treatment of patients with Cushing's disease; ClinicalTrials.gov identifier: NCT02180217), enrolled 137 patients with CD, of whom 120 had undergone pituitary surgery and 22 had undergone pituitary irradiation.^3^ The primary endpoint was the proportion of patients, randomly assigned to treatment in the randomized withdrawal period, who maintained mean UFC≤ULN, defined as complete response, to osilodrostat therapy or matching placebo at the end of the 8-week randomized withdrawal period (Period 3, Week 34), without any dose increase during this period. At the end of the randomization withdrawal period of 8 weeks, 31 (86.1%) of the 36 patients randomly assigned to continue osilodrostat, compared with 10 (29.4%) of the 34 patients randomly assigned to placebo, maintained a complete response.^3^

Beyond these data expressed in terms of primary endpoint, osilodrostat (at dosages of 4–60 mg/day) after 24 weeks of open-l abel treatment, induced, (considering the total population of 137 patients) a complete response in 72 (52.6%) patients without dose up-titration, and in 93 (67.9%) patients regardless of dose up-titration.^3^ Moreover, at Week 48, osilodrostat induced a complete response in 91 (66.4%) patients regardless of dose up-titration and a partial response in 13 (9.5%) patients, with an overall control in 75.9% of patients. The treatment with osilodrostat was associated with improvement in body weight, blood pressure, glucose metabolism, lipid profile, quality of life and psychological status. Focusing on the androgen production in females, at Week 48, mean serum testosterone levels increased from the normal range at baseline to the ULN, and hirsutism and acne were reported in 12 (11.3%) females. Whereas, in males, mean serum testosterone levels increased from the LLN at baseline to the mid-normal range at Week 48. The LINC 3 study also evaluated the impact of osilodrostat on pituitary tumour size. When considering patients with a measurable pituitary tumour both at baseline and at follow-up visit, an increase or decrease in tumour volume of ≥20.0% was observed in 30.3–37.5% and 28.8–32.8% of patients, respectively, after 24–48 weeks. Regarding the safety profile, nausea (41.6%), headache (33.6%), fatigue (28.5%) and AI (27.7%) were the most frequently reported AEs. AEs were also grouped into categories of special interest, including: hypocortisolism-related AEs (51.1%); adrenal hormone precursor accumulation-related AEs (42.3%), mainly represented by hypokalaemia (13.1%) and hypertension (12.4%); QT prolongation (3.6%); pituitary tumour enlargement (2.9%); and arrhythmogenic-potentially-related AEs (0.7%). Treatment discontinuation was reported in 24 (17.5%) patients as a result of: AEs in 15 (10.9%) cases, consent withdrawal in four (2.9%) cases, physician decision in three (2.2%) cases and patient or guardian decision in two (1.5%) cases.^3^

The optional extension phase of the LINC 3 study (ClinicalTrials.gov identifier: NCT02180217) enrolled 106 patients with CD, who at Week 48 were considered by study investigators to be receiving clinical benefit from treatment.^6^ The primary endpoint was the complete response or the partial response at Week 72. At Week 72, osilodrostat (at dosages of 4–60 mg/day) induced a complete response in 86 (81.1%) and a partial response in eight (7.6%) of 106 patients, with an overall control in 88.7% of patients. The observed improvement in body weight, blood pressure, glucose metabolism, lipid profile, quality of life and psychological status observed at the end of the core study was maintained or further improved during the extension period. Focusing on the androgen production in females, mean serum testosterone levels, which increased during the core period, decreased to within the baseline normal range, with no new cases of hirsutism reported, and an improvement of hirsutism severity. Stabilization or improvement occurred in females with both normal and elevated testosterone levels during the study; in males, no significant changes were observed compared with the end of the core period. Regarding the impact of osilodrostat on pituitary tumour size, and considering patients with measurable pituitary tumour at baseline and at least one post-baseline assessment, an increase or a decrease in tumour volume of ≥20.0% was observed in 38.9% and 29.6% of patients, respectively, from the core study baseline to Week 72. Considering the entire study, including the core and the extension periods, nausea (45.3%), headache (36.5%), fatigue (32.8%) and AI (29.2%) were the most frequently reported AEs. AEs were also grouped into categories of special interest, including: adrenal hormone precursor accumulation-related AEs (58.4%), mainly represented by hypertension (17.5%), peripheral oedema (16.1%), hypokalaemia (13.1%) and increased blood testosterone (11.7%); hypocortisolism-related AEs (54.0%); and pituitary tumour enlargement (16.1%); while AEs potentially related to arrhythmogenic potential and QT prolongation remained infrequent throughout the study. Treatment discontinuation was reported in 34 (32.1%) patients as a result of: AEs in 12 (11.3%) cases, patient or guardian decision in 12 (11.3%) cases, physician decision in five (4.7%) cases, consent withdrawal in two (1.9%) cases, death unrelated to osilodrostat treatment in two (1.9%) cases and unsatisfactory therapeutic effect in one (1.0%) case.^6^

#### The LINC 4 phase III study

LINC 4, the second phase III, multicentre, randomized study using both open-l abel and a double-blind, placebo-controlled methods (Efficacy and safety evaluation of osilodrostat in Cushing's disease; ClinicalTrials.gov: NCT02697734) enrolled 73 patients with CD, of whom 64 had undergone pituitary surgery and nine had undergone pituitary irradiation.^4^ The primary endpoint was the proportion of randomized patients with mean UFC≤ULN, defined as complete response at Week 12. At the end of the 12-week randomized withdrawal period, osilodrostat (at dosages of 4–60 mg/day) induced a complete response in 37 (77.1%) of the 48 patients randomly assigned to receive osilodrostat, compared with two (8.0%) of the 25 patients randomly assigned to receive placebo. Moreover, at Week 36, osilodrostat maintained a complete response in 59 (80.8%) of 73 patients. At Week 48, osilodrostat maintained a complete response in 50 (68.5%) and a partial response in eight (11.0%) of 73 patients, with an overall control in 79.5% of patients. The treatment with osilodrostat was associated with improvement in body weight, blood pressure, glucose metabolism, lipid profile, quality of life and psychological status. Focusing on the androgen production, serum testosterone levels increased in 18 (29.5%) females during the entire study, with hirsutism reported in seven (11.5%) females. Acne was reported in 10 (13.7%) patients. The LINC 4 study also evaluated the impact of osilodrostat on pituitary tumour size. When considering patients with a measurable pituitary tumour both at baseline and Week 48, an increase or decrease in tumour volume of ≥20.0% was observed in 40.0% and 28.6% of patients, respectively. Regarding the safety profile, decreased appetite (45.2%), arthralgia (45.2%), fatigue (38.4%) and nausea (37.0%) were the most frequently reported AEs. AEs were also grouped into categories of special interest, including: adrenal hormone precursor accumulation-related AEs (61.6%), mainly represented by increased blood testosterone (24.7%) and hypertension (21.9%); hypocortisolism-related AEs (27.4%); QT interval prolongation and/or arrhythmogenic potential (4.1%). Treatment discontinuation was reported in eight (11.0%) patients as a result of: patient/guardian/physician decision in five (6.9%) cases and AEs in three (4.1%) cases.^4^

### Studies in patients with Cushing's syndrome

#### A phase II study

Osilodrostat treatment has been investigated in the phase II, single-arm, open-l abel, multicentre Japanese study (Study of efficacy and safety of osilodrostat in Cushing's syndrome; ClinicalTrials.gov: NCT02468193), which enrolled nine patients with CS: five with adrenal adenoma, three with ectopic CS and one with macronodular adrenal hyperplasia.^21^ The primary endpoint was percentage change in mean UFC at the individual patient level from baseline to Week 12. At Week 12, osilodrostat (at dosages of 4–60 mg/day) induced a complete response in six (66.7%) and a partial response in one (11.1%) of nine patients, with an overall control in 77.8% of patients. Moreover, at Week 24, osilodrostat induced a complete response in two (22.2%) and a partial response in one (11.1%) of nine patients, with an overall control in 33.3% of patients. At Week 48, osilodrostat induced a complete response in one (11.1%) and a partial response in one (11.1%) of nine patients, with an overall control in 22.2% of patients. The treatment with osilodrostat was associated with improvement in waist circumference, blood pressure, glucose metabolism and lipid profile. Regarding the safety profile, AI (77.8%), gamma-glutamyl transferase increase (33.3%), malaise (33.3%) and nasopharyngitis (33.3%) were the most frequently reported AEs. AEs were also grouped into categories of special interest, including: hypocortisolism-related AEs (77.8%); adrenal hormone precursor accumulation-related AEs (33.3%), mainly represented by hypokalaemia (22.2%) and weight increase (11.1%). Treatment discontinuation was reported in six (66.7%) patients as a result of: AEs in four (44.5%) cases and patient/guardian decision in two (22.2%) cases.^21^

#### A case series based on real-life experience

Osilodrostat treatment has been investigated in a French case series, which was based on real-l ife experience that includes seven patients with CS due to adrenocortical carcinoma.^22^ Particularly, within 2 weeks, osilodrostat (at dosages of 2–60 mg/day) induced a significant decrease in UFC in six (85.7%) of seven patients, with control of hypercortisolism obtained within: 1 week in two (28.6%) patients, within 1 month in one (14.3%) patient, around 2 months in two (28.6%) patients and 3 months in two (28.6%) patients. The treatment with osilodrostat was associated with improvement in clinical picture, blood pressure and glucose metabolism. Regarding the safety profile, AI (42.9%) and worsening of hypokalaemia (14.3%) were the reported AEs. No treatment discontinuation was reported.^22^

### Studies in both patients with Cushing's syndrome and patients with Cushing's disease

#### A monocentre cohort study

Osilodrostat treatment has been compared with metyrapone treatment in a retrospective monocentre cohort study, which enrolled 16 patients with CS, seven with CD, five with ectopic CS, two with adrenal adenoma and two with adrenocortical carcinoma; eight were treated with osilodrostat (five with CD, one with ectopic CS, one with adrenal adenoma and one with adrenocortical carcinoma) and eight with metyrapone (two with CD, four with ectopic CS, one with adrenal adenoma and one with adrenocortical carcinoma).^23^ The primary aim was to compare the short-term efficacy of osilodrostat and metyrapone on mean UFC at baseline and at Weeks 2, 4 and 12. At Week 2, osilodrostat (at dosages of 3–7 mg/ day) and metyrapone (at dosages of 500–2000 mg/day) induced a mean UFC decrease from baseline of 68.4% and 21.3%, respectively. At Week 4, osilodrostat (at dosages of 4–20 mg/day) and metyrapone (at dosages of 500–2000 mg/day) induced a mean UFC decrease from baseline of 50.1% and 37.3%, respectively. Particularly, considering patients reaching Week 4, osilodrostat induced a complete response in three (42.9%) of seven patients, whereas metyrapone did not induce complete response in any of the six patients. At Week 12, osilodrostat (at dosages of 6–10 mg/day) and metyrapone (at dosages of 1000–2000 mg/day) induced a mean UFC decrease from baseline of 51.5% and 71.5%, respectively. Particularly, considering patients reaching Week 12, osilodrostat induced a complete response in two (50.0%) of four patients, whereas metyrapone induced a complete response in two (66.7%) of three patients. At Week 4, the treatment with osilodrostat was associated with improvement in systolic and diastolic blood pressure, whereas the treatment with metyrapone was associated with no relevant changes in systolic blood pressure and with worsening in diastolic blood pressure. Although both drugs showed comparable efficacy, osilodrostat reduced cortisol levels and controlled blood pressure faster. Regarding the safety profile, hypokalaemia (50.0%), depression (12.5%), asthenia (12.5%), nausea (12.5%) and QT prolongation (12.5%) were the most frequently reported AEs during osilodrostat treatment. Hypokalaemia (37.5%), asthenia and dizziness (25.0%) were the most frequently reported AEs during metyrapone treatment. During osilodrostat treatment, discontinuation was reported in four (50.0%) patients as a result of: tumour resection in two (25.0%) cases, AEs in one (12.5%) case and failure to attend follow-up in one (12.5%) case. During metyrapone treatment, discontinuation was reported in five (62.5%) patients as a result of: AEs in two (25.0%) cases, failure to attend follow-up in one (12.5%) case and unknown reasons in two (25.0%) cases.^23^

No clinical studies have been performed on osilodrostat use in combination with different agents. However, anecdotal cases have shown that osilodrostat, in combination with etomidate in a patient with severe adrenocorticotropic hormone (ACTH)-dependent CS, as well as in combination with ketoconazole in a patient with ACTH-independent CS, was highly and rapidly effective, with a significant improvement in clinical picture, while being well-tolerated.^6,24,25^

## Discussion

Based on relevant efficacy data, the adrenal-directed drug osilodrostat appears able to induce a potent, rapid and effective hypercortisolism control, and to maintain sustained efficacy in the long-term follow-up, without reported escape phenomenon. Therefore, it represents a promising drug for the treatment of CS.

Particularly, osilodrostat can be considered a valid medical option in cases of severe disease and/or in cases requiring a prompt relief from hypercortisolism-related comorbidities.^7–9^ Moreover, osilodrostat can be considered in cases of surgery failure for persistent or recurrent CS, in cases where surgery is not an option or refused, or in cases with failed previous medical attempts.^7,8^

The open-label, non-randomized, phase II trials, the double-blind, randomized, phase III trials, and the case series demonstrated that osilodrostat (at dosages of 2–100 mg/day), administered in 387 patients (368 with CD, four with ectopic CS and 15 with adrenal CS), appears associated with improvement in cardiovascular and metabolic parameters, and quality of life.^1–6,21–23^ Particularly, blood pressure, body weight, waist circumference, glucose metabolism, lipid profile, quality of life and psychological status have ameliorated.^1–6,21–23^

Focusing on the beneficial effects of osilodrostat on blood pressure, a direct effect of osilodrostat itself, and a direct role of cortisol lowering on vessels have been hypothesized as the major putative contributing factors, possibly overcoming the potential hypertensive effects of mineralocorticoid precursor accumulation.^18,26,27^

Focusing on lipid profile, a favourable effect in total cholesterol, low-density lipoprotein cholesterol and triglycerides has been observed.^2–4,6,21^ Contrasting data have been reported in high-density lipoprotein cholesterol, with increased or decreased levels, although the mechanism appears to be unknown.^2–4,21^

Notably, considering that the addition or the adjustment of concomitant medications for concomitant comorbidities, such as anti-hypertensives, anti-diabetic and anti-dyslipidaemic drugs, were permitted during the studies, it cannot be excluded that these drug changes may at least partially have contributed to the observed improvements.^1–3^

Overall, although the reported improvements may be most attributable to disease control, a direct role of osilodrostat itself and/or of concomitant drug changes cannot be excluded, mainly considering that some reported improvements occurred irrespective of cortisol control.

Compared with metyrapone and ketoconazole, osilodrostat, due to its higher potency and longer half-l ife with a twice daily administration schedule, putatively permits relatively lower dosages to achieve the same efficacy with a potential reduction in the AEs rate. Furthermore, it favours treatment compliance, consequently inducing a possible positive impact on the success rate.^7–9^

To individualize the most effective dosage, an initial frequent monitoring every 1–2 weeks, and thereafter at least every 2 months, is required, unless a different monitoring is recommended on the basis of individual clinical response and tolerability.^15–18^ Based on the individual response and tolerability, the timing of the dose may also be individualized; for example, in cases of insomnia, a higher dose of osilodrostat in the evening may be hypothesized, aimed at improving the normal cortisol circadian rhythm and optimizing the hypercortisolism control.^28^

Osilodrostat appears to be a promising drug when considering its safety profile, characterized by the occurrence of common AEs including decreased appetite, nausea, diarrhoea, fatigue, headache and arthralgia.^1–4,6,1–6^ However, some specific safety issues should be considered in clinical practice and herein will be discussed.

Osilodrostat, potently and rapidly blocking adrenal enzymes, may induce hypocortisolism-related AEs. Patients may develop AEs characterized by low cortisol levels, which may be associated with hypotension and hypoglycaemia, and a clinical syndrome suggestive of hypocortisolism; this clinical syndrome mainly includes fatigue, dizziness, decreased appetite, nausea, vomiting, diarrhoea and abdominal pain, up to the occurrence of syncope and adrenal crises.^28^ This hypocortisolism condition should be differentiated from glucocorticoid withdrawal syndrome, which may appear with similar signs and symptoms, although generally less severe, with a reduction in cortisol levels that were previously pathologically elevated in the absence of low cortisol values in the morning, hypotension and hypoglycaemia.^28^ Notably, in at least some cases of “suggestive” signs and symptoms, frequently described as isolated and not always categorized as hypocortisolism-related AEs, a condition of hypocortisolism or of glucocorticoid withdrawal syndrome cannot be excluded.

Noteworthy, in the measurement of cortisol, it is recommended to use laboratory methods that do not exhibit significant cross-reactivity with cortisol precursors, such as 11-deoxycortisol, which may increase during osilodrostat treatment and be potentially responsible of cortisol overestimation. Therefore, mass spectrometry assays that exhibit a high degree of specificity for steroid hormone measurement should be preferred over immunoassays.^28^ Although all approved immunoassays may be susceptible to interferences causing false results, the most recent immunoassays seem to present a less relevant cross-reactivity, which requires caution in the interpretation of the results.^29^ In case of discordant results between biochemical and clinical picture, a further evaluation should be performed or an alternative method of measurement should be selected.

In cases of stress conditions, such as fever, infections, physical and emotional stress, or acute illness, and in situations where the hypothalamus-hypophysis-adrenal axis is physiologically activated, signs and symptoms of hypocortisolism should be more frequently and deeply monitored due to the higher risk of developing hypocortisolism.^28^

Notably, a more frequent and careful clinical monitoring, particularly in the first weeks of treatment, is also suggested in: cases of mild hypercortisolism characterized by a mild elevation of cortisol levels, due to the higher risk of developing hypocortisolism; and cases of severe hypercortisolism characterized by a severe elevation of cortisol levels, due to the higher risk of developing the glucocorticoid withdrawal syndrome, and due to the higher starting doses and faster up-titrations that may have been necessary to obtain a more rapid hypercortisolism control.^28,30^

Due to the risk of developing hypocortisolism-related AEs, clinicians should: adequately educate all patients treated with osilodrostat on their condition; inform patients on how to recognize signs and symptoms of hypocortisolism; train patients regarding stress-doses, intercurrent illnesses and emergency glucocorticoid administration; recommend vaccinations; and suggest patients carry on their person, an emergency card/bracelet/necklace regarding the hypocortisolism condition.^28^

In cases of mild hypocortisolism, temporary dose reduction of osilodrostat for a few days may improve signs and symptoms while follow-up biochemical evaluation is performed. In cases of moderate to severe hypocortisolism, temporary or definitive interruption of osilodrostat can be considered and, if necessary, glucocorticoid replacement therapy should be initiated.^28^ Particularly, in cases of severe hypocortisolism, the doses of glucocorticoid replacement therapy should be higher, up to five times the usual doses, and if severe diarrhoea or vomiting occur, the oral glucocorticoid regimen should be replaced by a parenteral one and an access to an emergency unit should be suggested.^28^

In cases of severe disease characterized by a severe elevation of cortisol levels, the gradual dose up-titration, although potentially useful to prevent the glucocorticoid withdrawal syndrome, may be inadequate to obtain a rapid control of hypercortisolism, and a starting treatment with higher doses may be required.^7^ This condition may benefit from a “block-and-replace” regimen, consisting of treating patients with a combined medical approach with osilodrostat and exogenous glucocorticoids; although this represents an empirical approach, not systematically used in clinical practice and not proven to be superior.^7,8,28^ This approach may induce a more rapid disease control with high doses of osilodrostat and contain the risk of developing hypocortisolism-related AEs, however, caution is required to avoid iatrogenic CS.^7,8,28^

Osilodrostat blocking adrenal enzymes may induce the accumulation of mineralocorticoid precursors and androgen excess due to pathway diversion, causing adrenal hormone precursor accumulation-related AEs, mainly represented by hypokalaemia, hypertension and peripheral oedema, hirsutism and acne. Particularly, due to the potential occurrence of hypokalaemia, osilodrostat, although not specifically recommended,^17,18^ should be preferred in patients without severe or with well-controlled hypokalaemia.^9^ Nevertheless, a close and careful monitoring of potassium levels is recommended, both before starting treatment, during osilodrostat dose changes and during the clinical follow-up, considering patients’ characteristics, disease, medical history, and especially risk factors for hypokalaemia.^7–9,28^ Oral potassium replacement, mineralocorticoid receptor antagonists (spironolactone or eplerenone), osilodrostat dose reduction or interruption, or a combination of these approaches may represent possible options to manage hypokalaemia.^28^ In cases of low-normal potassium levels and hypertension, mineralocorticoid receptor antagonists may be preferred; in cases of below normal potassium levels, oral potassium replacement is recommended; in cases of severe hypokalaemia, which are resistant to mineralocorticoid receptor antagonists and oral potassium replacement, osilodrostat interruption and intravenous potassium replacement may be required.^28^

In females, due to the potential occurrence of hirsutism, mainly due to hyperandrogenism, local treatment and spironolactone, with its androgen receptors blocking activity, may be added.^28^ Notably, the elevation of serum testosterone levels may be transient, and may spontaneously reduce despite osilodrostat treatment, along with an improvement in hirsutism.^6^ The reduction in testosterone levels towards normal ranges may be at least partially due to the lower long-term osilodrostat maintenance doses, and due to benefit from hypercortisolism control.^6^ However, in cases of persistent/worsened hirsutism and permanent hyperandrogenism, an alternative CS medical treatment with a different mechanism of action may be considered.^28^

Conversely, in males, testosterone levels increase towards normal ranges.^2,3^ This may be due to the benefit of hypercortisolism control, which induces the reversal of gonadotropins blockade caused by chronic hypercortisolism.^31^

Due to the potential occurrence of prolonged QT interval, monitoring potassium and magnesium levels, together with the electrocardiogram (ECG) evaluation, before starting treatment and during the clinical follow-up, is strongly recommended.^28^ Particularly, ECG should be performed at baseline and 1 week after initiating osilodrostat treatment to ensure that there is no QT prolongation. Thereafter, ECG should be repeated after 2–4 weeks of treatment, depending on age and cardiac history, and in cases of concomitant medications known to prolong the QT interval and induce electrolyte abnormalities.^28^

Lastly, regarding the impact of osilodrostat on pituitary tumour size, similar proportions of patients had either an increase or decrease in tumour size.^3,4^ However, all patients treated with osilodrostat should undergo imaging procedures for corticotroph tumour progression, due to the potential risk of pituitary tumour enlargement. Notably, it has not been demonstrated if the risk of this enlargement during medical therapy may be related to the medical therapy itself, or would have occurred also in the absence of medical therapy due to the disease natural history.^28^

Particularly, a potential sign of tumour enlargement may be represented by the progressive elevation in ACTH levels, although they do not appear to linearly correlate with tumour progression, due to the short half-l ife and fluctuation of ACTH levels.^28^ However, the increase in ACTH levels, especially if substantial and rapid, may represent an alert to promptly perform imaging procedures.^28^

A magnetic resonance imaging (MRI) before treatment at baseline, followed by regular serial MRIs, are advised to monitor pituitary tumour size in most patients.^7,28^ In cases lacking evidence of a pituitary tumour at baseline, the subsequent MRI should be performed after 1–2 years, or sooner in cases of substantial and rapid increase of ACTH levels. If there is evidence of a pituitary tumour at baseline, the subsequent MRI should be performed after 6–12 months. If a tumour size progression is observed, osilodrostat treatment interruption should be evaluated, and a neurosurgical consultation is recommended.^28^

## Conclusion

Treatment with osilodrostat has acquired an emerging role in the medical management of CS, especially thanks to its rapidity of response, easy use, relevant biochemical and clinical efficacy and favourable safety. Overall, osilodrostat may have the appropriate profile to tailor therapy on a patient-b y-patient basis, trying to fill some of the still unmet needs in the management of CS. Further real-world clinical experience data are needed to confirm the current knowledge.
